# EHMTI-0166. Chronic daily headace-impact of adverse childhood experiences

**DOI:** 10.1186/1129-2377-15-S1-D2

**Published:** 2014-09-18

**Authors:** M Bøe, ET Thortveit, A Vatne, I Frøytland, LK Kjellevold, K Stø, A Mygland

**Affiliations:** 1 Neurological Department, Sørlandet hospital Kristiansand, Kristiansand, Norway

## Background

In “Kronhodestudien” (KS) published in 2007-2009 we followed a cohort of 80 patients with chronic daily headache one year after withdrawal therapy and found that headache improved by > 50% in a third, and a third did not improve [1].

## Aim

To examine whether adverse childhood events is associated with chronic headache and prognosis after withdrawal therapy.

## Method

All 80 patients from the KS study were invited to a follow-up in 2013. Headache was registered, and a questionnaire concerning Adverse Childhood Events (ACE) was delivered to KS patients and controls without headache or other chronic pain. The study was performed anonymously and approved by the ethical committee.

## Results

Sixty-six KS patients (73% women) and 69 controls (70% women) were included. Total ACE score was higher in patients [1.6 (1.1-2.1)] than in controls [0.9 (0.5-1.3)], p=0.02. Reporting sexual violation was more common in patients without improvement of headache [9/24 (38%)] than in controls [7/69 (10%)], p <0.01. An ACE score > 4 was more common in patients who did not improve [7/24 (29%)] than in patients who had improved more that 50% [1/21(5%), p 0.05], and more common than in controls [8/69(12%), p=0.04].

## Conclusion

Adverse childhood experience seems to be more common in patients with chronic headache than in controls, and most common in patients with a poor response to withdrawal therapy.

No conflict of interest.
